# Supplementary material for: A light-activatable theranostic combination for ratiometric hypoxia imaging and oxygen-deprived drug activity enhancement
Source: Nat Commun. 2024 Jan 2;15:153. doi: 10.1038/s41467-023-44429-y (PMC10762052; doi:10.1038/s41467-023-44429-y)
Supplement: Supplementary file 1 — Supplementary Information [file 41467_2023_44429_MOESM1_ESM.pdf]

Supplementary Information for

A Light-Activatable Theranostic Combination for Ratiometric Hypoxia Imaging and Oxygen-Deprived Drug Activity Enhancement

Lei Ge[1], Yikai Tang[1], Chongzhi Wang[1], Jian Chen[1], Hui Mao[2], Xiqun Jiang[1]*

[1]College of Chemistry and Chemical Engineering, Nanjing University, Nanjing 210023, China

[2]Department of Radiology and Imaging Sciences, Emory University, Atlanta GA 30322, USA

*Correspondence to: jiangx@nju.edu.cn;

Contents:

1. Supplementary Methods……………………………………………………………….3

2. Supplementary Figures……………………………………………………………….13

3. Supplementary Table……………………………………………………………………46

Supplementary Methods

Materials

Phenylacetic acid, zinc chloride, phthalimide, hydrochloride acid, zinc phenylacetate, α, ω-amino-terminated polyethylene glycol (PEG, *Mw* = 2 kDa), Chloro (1,5-cyclooctadiene) iridium(I) dimer ($[Ir(COD)_2Cl_2]_2$), Chlorosulphonic acid were obtained from J & K Chemical Ltd. Tirapazamine (TPZ), AQ4N, PR104 and TH302 and solvents were purchased from Sigma-Aldrich Chemical Co. All the commercially available reagents were used as received without any further purification. The cell culture products were purchased from Thermo Fisher Scientific unless otherwise stated.

Preparation of IrTBP

The preparation of IrTBP followed a reported method. Zinc meso-tetraphenyltetrabenzoporphyrin (ZnTBP) was prepared according to previous report by phthalimide, phenylacetic acid and zinc phenylacetate. The phthalimide (2.94 g, 20.0 mmol), phenylacetic acid (3.60 g, 26.6 mmol) and zinc phenylacetate (1.68 g, 5.0 mmol) were added into a 100 mL Schlenk flask filled with argon. The mixture was heated and stirred at 360 °C for 1 h. The mixture was cooled, dissolved in acetone, precipitated with DI water and washed three times. The crude product was dissolved in toluene and purified on neutral alumina column. The column was eluted with hexane/toluene (2:1, v/v) firstly and then with toluene to remove the yellow and the red fractions, respectively. Finally, dichloromethane/ tetrahydrofuran (98.5:1.5, v/v) was used to elute the product. Yield: 736.1 mg (16.8%). ^1H NMR (400 MHz, DMSO-d6): δ 8.27 (d, J = 7.1 Hz, 8H), 8.03 (d, J = 6.8 Hz, 4H), 7.95 (t, J = 7.3 Hz, 8H), 7.30 – 7.24 (m, 8H), 7.09 – 7.06 (m, 8H). MS (MALDI): calc. [M]+ m/z 876.223, found 876.716.

Next, to synthesis meso-tetraphenyltetrabenzoporphyrin (H_2TBP), ZnTBP (500.0 mg, 0.57 mmol) prepared was dissolved in 200 mL dichloromethane and 150 mL hydrochloric acid solution (30% HCl). The solution was stirred for 1h at room temperature. After that, 1000 mL DI water was added into the solution and extracted with dichloromethane (3×300 mL). The organic layer was collected and the resulting solid was washed three times and dried. The crude product was dissolved in

dichloromethane and purified by silica gel column chromatography using dichloromethane as the eluent agent to give dark-green powder. Yield: 159.5 mg (34.3%). ^{1}H NMR (400 MHz, DMSO-d6): δ 8.31 (d, J =5.9 Hz, 8H), 8.02 (t, J = 7.4 Hz, 4H), 7.93 (t, J = 14.1 Hz, 8H), 7.37 – 7.11 (m, 16H), -1.10 (s, 2H). MS (MALDI): calc. [M]+ m/z 814.310, found 816.038.

H_2TBP (100 mg, 0.12 mmol) and chloro (1,5-cyclooctadiene) iridium(I) dimer ([Ir(COD)$_2$Cl$_2$]$_2$, 125 mg, 0.186 mmol) were dissolved in 80 mL ethylene glycol and stirred at 170 ^{o}C for 6 h. After completion of the reaction, 100 mL DI water was added into the mixture. The precipitate was collected and washed three times. The dried crude product was dissolved in acetone and purified by chromatography using a neutral alumina column. The column was eluted with toluene/acetone (1:1, v/v) firstly to remove the impurities and then eluted with acetone/methanol (95:5, v/v) to obtain Iridium meso-tetraphenyltetrabenzoporphyrin (IrTBP). Yield: 51.7 mg (55.3%). ^{1}H NMR (400 MHz, DMSO-d6) δ 8.26 (d, J = 7.5 Hz, 8H), 8.03 (d, J = 7.6 Hz, 4H), 7.94 (t, J = 7.5 Hz, 8H), 7.29 – 7.22 (m, 8H), 7.10 – 7.03 (m, 8H). MS (MALDI): calc. [M]+ m/z 1005.273, found 1005.950.

Preparation of Ir-NP

The above prepared IrTBP (50.0 mg, 0.05 mmol) was added into 7 mL chlorosulfonic acid and stirred overnight at room temperature for 12 h. After completion of the reaction, the mixed solution was added slowly dropwise to 100 mL saturated NaCl solution at -5 ^{o}C and filtered quickly. The precipitate was washed with ice water and the resulting IrTBP-chlorosulfonate was dried in an oven with no more purification. And the tetraphenylporphyrin (TPP)-chlorosulfonate was prepared with a similar procedure.

The fresh prepared IrTBP-chlorosulfonate (10.0 mg) and TPP-chlorosulfonate (40.0 mg) were added into a flask with dichloromethane (10.0 mL). After stirred 5 min and dissolved, the mixture was added into α, ω-amino-terminated polyethylene glycol (PEG, Mw = 2 kDa, 500.0 mg) and pyridine (0.05 mL) as catalytic. After stirred at room temperature for 3 d, the solvent was removed by centrifugation and the residue was

added into 30 mL DI water. After 30 min sonication and filtration followed, the filtrate was dialyzed (molecular weight cut off (MWCO) 3500 Da) for one week with DI water. The product was finally obtained by lyophilization.

Optical properties of Ir-NP

UV-Vis spectrometer (Shimadzu UV-2401) were used to study the absorption of Ir-NP. The emission of Ir-NP was measured by luminescence spectrometer (HORIBA FM-4NIR) and the excitation wavelength was 620 nm. To investigate the effect of different oxygen levels to the emission of Ir-NP, different ratios of oxygen-nitrogen gas mixture (0%, 10%, 21%, 50%, 75%, 100% O_2) were introduced into the solution using a gas flow meter for 5 min each time to saturate followed the measurement.

Luminescence imaging of Ir-NP

Ir-NP was prepared in aqueous solutions of 0.1, 0.2, 0.5, 1, 2, 3 and 4 mg/L, respectively. The seven different concentrations of the Ir-NP were placed under three different oxygen levels (0%, 6% and 21% O_2, created by Anaero Pack-Anaero). After 30 min, the luminescence imaging windows at 675 ± 25 nm and 775 ± 25 nm were obtained with the imaging system, respectively. The excitation light source was a xenon lamp at 605 nm, and the ratio-metric imaging were calculated by ImageJ software.

Cell uptake

4T1 cells were inoculated in 6-well plates, and 200 μL of 50 mg/L Ir-NP solution was added and incubated for 4 h. Next, the cells were fixed with 4% paraformaldehyde and DAPI was added to stain the cell nuclei. The intracellular fluorescence distribution was subsequently observed on a confocal microscope (LSM 710). The imaging window of DAPI was set to 430 – 500 nm and the excitation wavelength was 405 nm. The imaging window of Ir-NP was set to 650 – 700 nm and the excitation wavelength was 630 nm.

Ratio-metric imaging of Ir-NP *in vitro*

4T1 cells were inoculated in three groups of 96-well plates, and 20 μL 50 mg/L Ir-NP solution was added to each group and incubated for 4 h. Each well was isolated from air by adding non-toxic oil (Enzo Life Sciences), and the incubation was continued

for 0 h, 6 h and 12 h, respectively. The luminescence imaging windows at 675 ± 25 nm and 775 ± 25 nm were obtained with the imaging system, respectively. The excitation wavelength was 605 nm.

To study the oxygen consumption rate of different cells, H22, 4T1 and Bend.3 cells were inoculated in three groups of 96-well plates, respectively. 20 μL 50 mg/L Ir-NP solution was added to each group and incubated for 4 h. Each well was isolated from air by adding non-toxic oil (Enzo Life Sciences), and the incubation was continued for 12 h. The luminescence imaging windows at 675 ± 25 nm and 775 ± 25 nm were obtained with the imaging system, respectively. The excitation wavelength was 605 nm.

Ratio-metric imaging of Ir-NP *in vivo*

100 μL of 4T1 cells (5×10^6) in PBS suspension was injected into the right posterior side of each BALB/c mouse to establish 4T1 tumours. BALB/c mice bearing 4T1 tumour were injected with Ir-NP (200 μL, 1 mg/mL) intravenously. The luminescence imaging windows at 675 ± 25 nm and 775 ± 25 nm were observed at different time. At the end of the *in vivo* imaging, the mice were sacrificed, and their major organs and tumours were imaged.

To study the real-time monitoring oxygen, 100 μL of 4T1 cells (5×10^6) in PBS suspension was injected into the left hind leg of BALB/c mice to establish tumours. Ir-NP (200 L, 1 mg/mL) was given intravenously into BALB/c mice with 4T1 tumour. After 24 h, the left hind leg of mice was ligatured to slow down the blood flow. After ten minutes, the bundle was released. The luminescence imaging is performed at 5-minute intervals throughout the procedure, lasting 30 minutes in total.

Biodistribution of Ir-NP

Saline and Ir-NP were injected into mice with subcutaneous 4T1 tumours intravenously. At different time points, mice were sacrificed and the tumours and major organs (including heart, liver, spleen, lung, kidney) were removed and weighed. Three samples were set up at each time point and the saline-injected mice were set as background group. 5 mL dichloromethane was added to each centrifuge tube as extraction solution. Subsequently, the tubes were homogenized thoroughly and

extracted for two days at room temperature. After the centrifugation and removement of the precipitate, the concentration of Ir-NP was determined by fluorescence spectrometry (Excitation: 635 nm. Emission: 665 nm). And the major organs: liver, heart, spleen, lung, kidney and the tumors were also collected, weighed, homogenized and then dissolved digested in *aqua regia*, followed by ICP-AES to determine the Ir concentration.

Singlet oxygen detection

1 mL 20 μM SOSG solution was added to 3 mL 5 mg/L Ir-NP aqueous solution. Followed by irradiation with a 635 nm light source, the fluorescence emission of SOSG at 525 nm was measured every 40 s with an excitation wavelength of 465 nm. At the same time, the rate of single-linear oxygen generation from Ir-NP at different oxygen concentrations was measured with similar procedure.

To detect the intracellular generation of ROS by Ir-NP, 4T1 cells were inoculated onto 6-well plates with samples and incubated for 4 h. After removing the medium and washing three times with PBS solution, 40 μM 2',7'-dichlorofluorescein diacetate (DCFH-DA) was added and incubated for 30 min. After the incubation, the cells were irradiated with 635 nm laser (50 mW cm^{-2}) for 3 min and observed on a confocal microscope (Excitation: 488 nm).

Preparation of COF@T

COF@T was prepared with previous report. In brief, benzidine (0.045 mmol, 8.3 mg) and 2,4,6-trihydroxybenzene-1,3,5-tricarbaldehyde (0.030 mmol, 6.3 mg) were dissolved in 100 mL of anhydrous ethanol and then stirred for 1 h at room temperature. After centrifugation, washing and drying, the obtained yellow solid was mixed with TPZ and mPEG-NH_2 (Mw = 5000) in DMSO. After stirred 12 h, the impurities were removed by dialysis and the product was obtained by lyophilization as powder, stored in -5 °C. The quantity of loaded drug was determined using a UV-vis spectrophotometer at a wavelength of 461 nm. The drug loading capacity were calculated using the following equations:

$$\text{Loading capacity} = \frac{\textit{Weight of drugs in NPs}}{\textit{Weight of NPs}} \times 100\%$$

The loading capacity of TPZ was calculated as 12.1%.

In vitro TPZ release of COF@T

A 1 mL volume of COF@T (10 mg/mL) was placed into a dialysis bag with a molecular weight cutoff of 12 kDa. The dialysis bag was then immersed in 5 mL of release medium (PBS, 0.01 M, pH = 7.4, containing 0.1% v/v Tween 80) and incubated at 37°C. At predetermined time intervals, the release medium was completely withdrawn and replaced with an equivalent fresh release medium. The TPZ content in the collected release medium was determined using fluorescence spectroscopy with excitation at 495 nm and emission at 590 nm.

Surface area and pore size of COF

Nitrogen adsorption experiments were conducted on an ASAP 2010 micropore physisorption analyzer (Micromeritics, Norcross, GA, USA) using adsorption at 77 K. The pore size distribution of the COF was determined using density functional theory (DFT) method.

Apoptosis assay

4T1 cells were inoculated onto 96-well plates at a density of 5000 cells per well, and after 24 h incubation, Ir-NP with various concentrations was added and incubated for 6 h followed by irradiation with a 635 nm laser (50 mW cm^{-2}) for 10 min, after which MTT solution (5 mg mL^{-1}) was added. After 4 h incubation, 100 μL aqueous solution containing 10 g sodium dodecyl sulfate, 5 mL isobutanol and 0.1 mL 10 M HCl was added to each well, followed by 24 h incubation before measuring the absorbance of thiazolyl blue MTT at 570 nm. Three samples were set up for each group of concentrations. Similar to the procedure, the MTT results of COF@T and COF@T combination with Ir-NP under different conditions (including normoxia, hypoxia and normoxia with light) were obtained.

To test the cytotoxicity of Ir-NP for normal cells, Bend.3 cells were used for MTT assay. The experimental procedure is similar to the above steps, with Bend.3 cells maintained under normoxia conditions.

Therapeutic effect of our theranostic system.

4T1 cells (5×10^6) in PBS suspension was injected into the right posterior side of BALB/c mouse to establish 4T1 tumours. When the tumour volume reached roughly 100 mm^3, the mice were divided into five groups (5 mice in each group): (1) PBS; (2) COF@T; (3) Ir-NP + hv; (4) Ir-NP + 3hv; (5) Regimen-1; (6) Regimen-2. All samples were administrated via tail vein, respectively. The dose of TPZ was 9 mg/kg and the dose of Ir-NP was 20 mg/kg. The tumour of mice in (3) and (5) was irradiated with 635 nm laser (150 mW cm^{-2}) for 15 minutes at 24 h after the injection. And the tumour of mice in (4) and (6) was irradiated with 635 nm laser (150 mW cm^{-2}) for 5 minutes at 24 h, 48 h and 72 h. The tumour volume and body weight of mice were measured on alternate day. The volume of tumour was calculated as follows:

$$Volume = 0.5 * a * b * b$$

In the equation, a represents the maximum diameter of tumour while b represents the minimum diameter of tumour. Hematoxylin and eosin (H&E) staining was carried for tumours and main organs harvested from each group at day 14.

Other hypoxia prodrugs

The preparation and other experiments of COF@AQ, COF@PR and COF@TH were conducted with COF@T similarly.

Statistical analyses

Error bars are reported as mean ± s.d. Differences between groups were compared by analysis of variance (ANOVA) and Student's t-test. A P-value < 0.05 was considered to be statistically significant.

Supplementary Figures

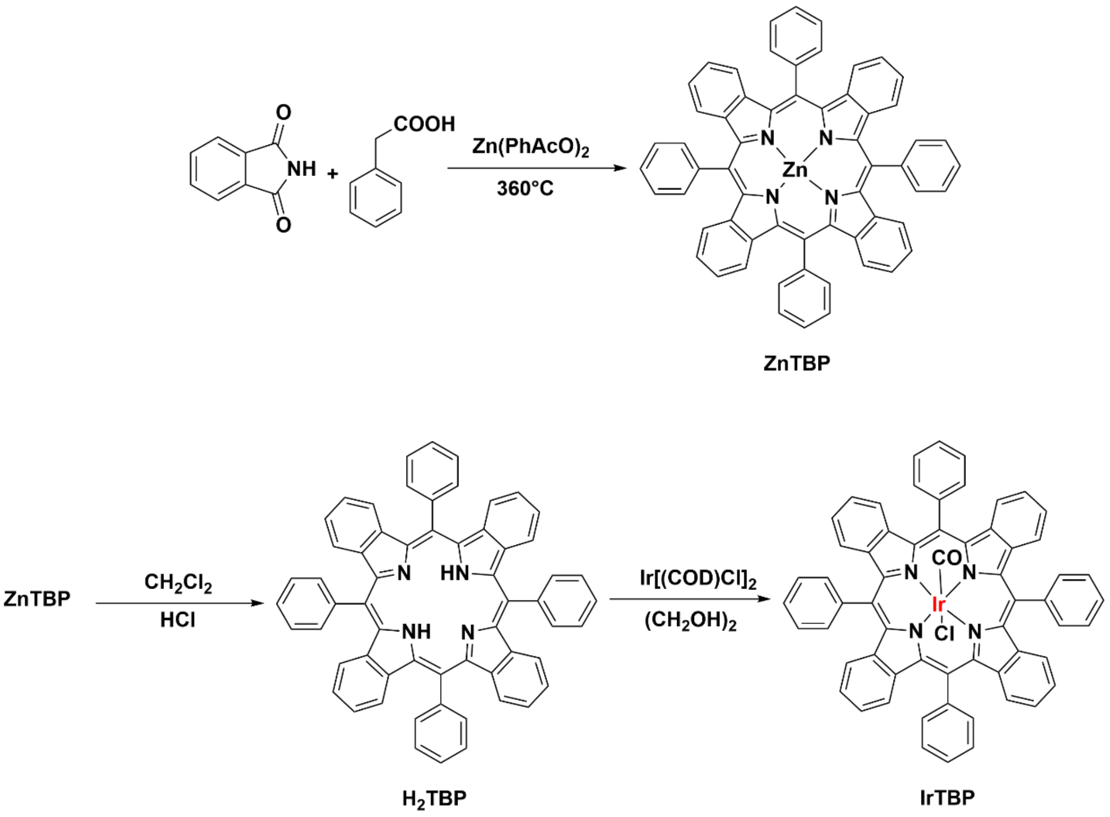

Supplementary Fig. 1. The synthesis route of IrTBP.

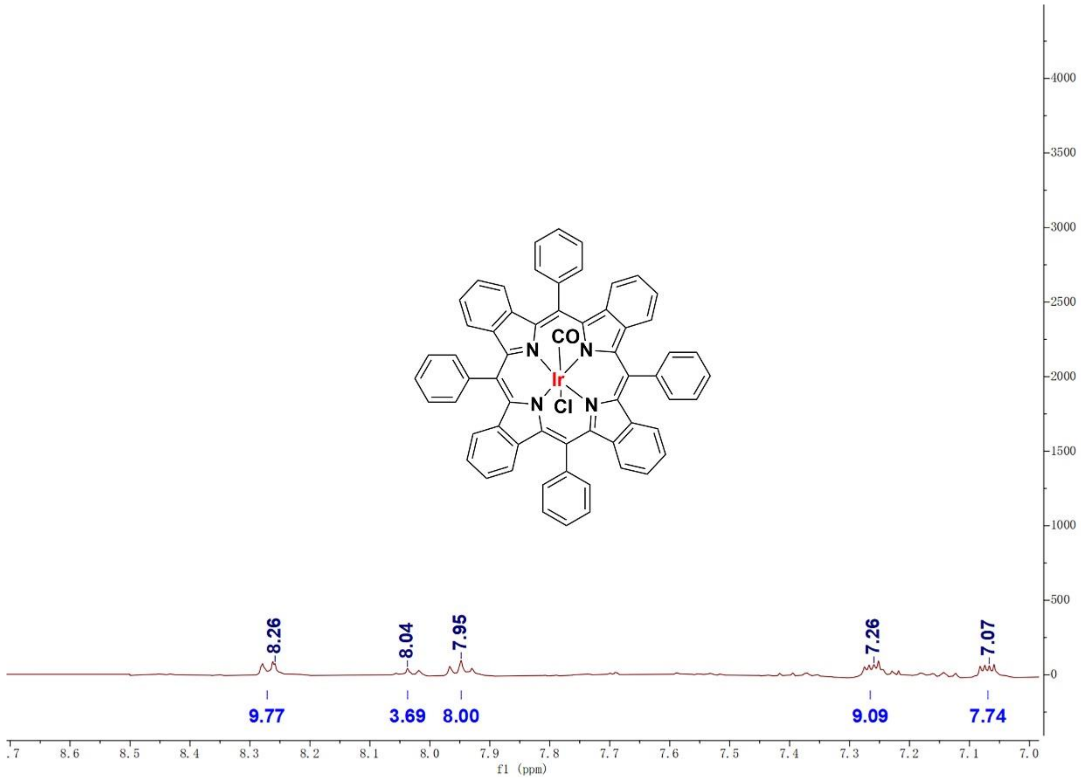

Supplementary Fig. 2. The ^{1}HNMR spectrum of IrTBP.

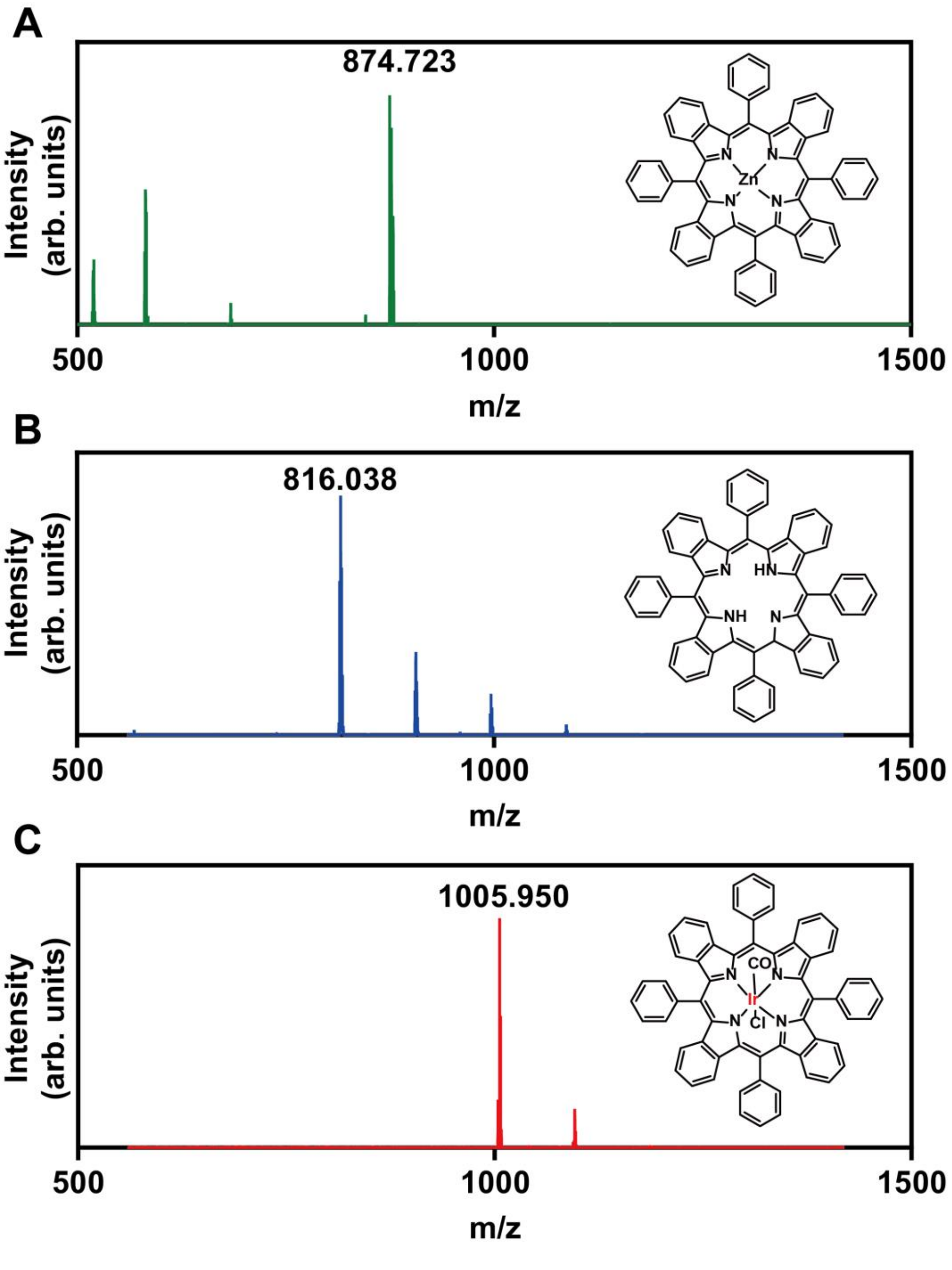

Supplementary Fig. 3. The MALDI-HRMS of ZnTBP (A), H_2TBP (B) and IrTBP (C). The term (arb. units) is abbreviated for arbitrary units.

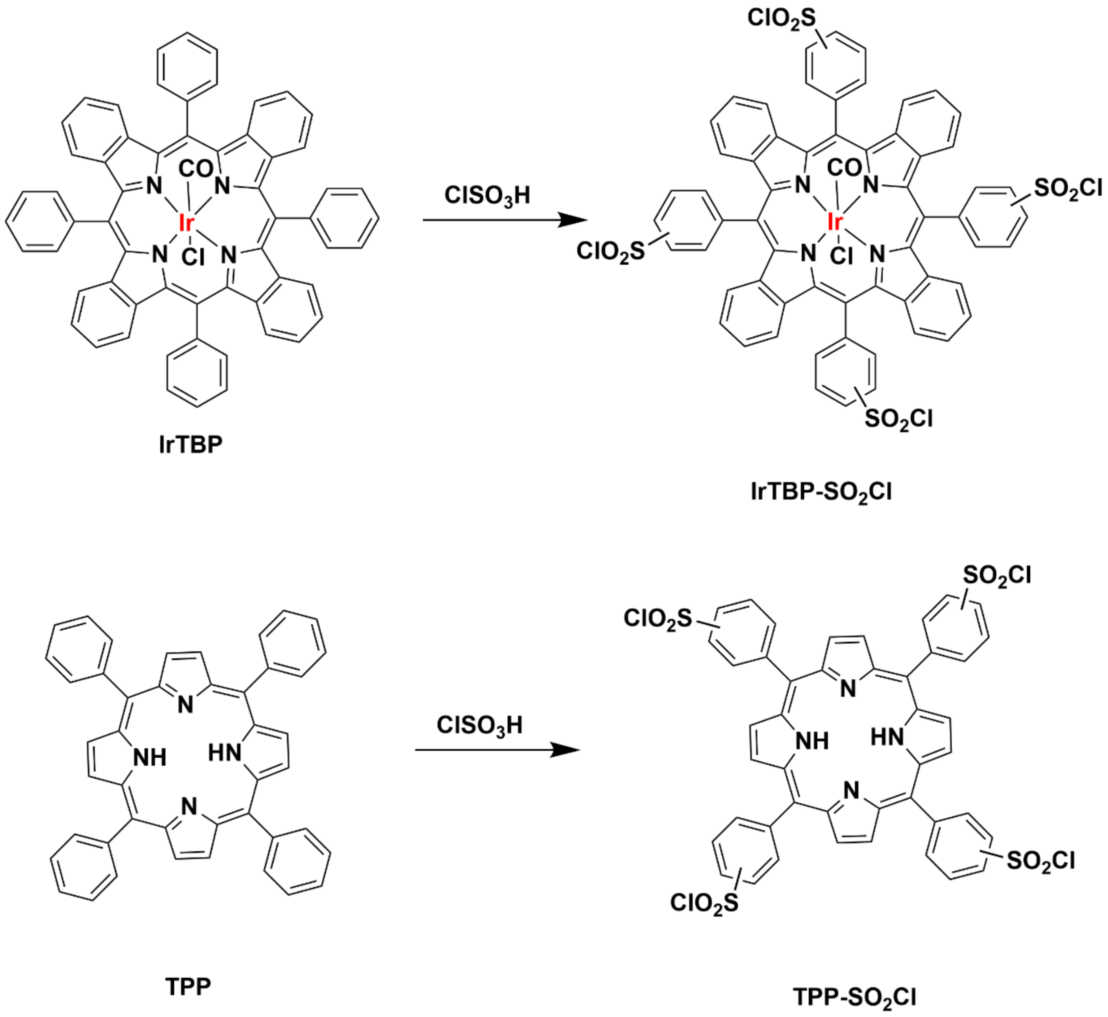

Supplementary Fig. 4. Chlorosulfonylated of IrTBP and TPP.

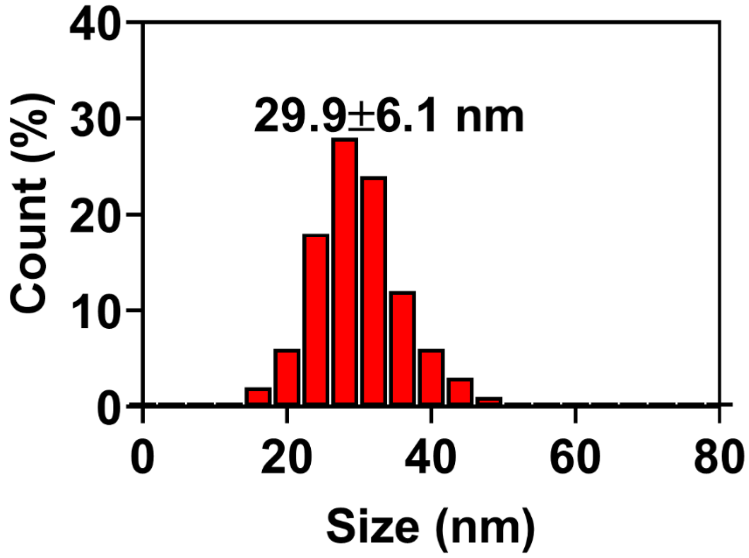

Supplementary Fig. 5. The size distribution of Ir-NP counted in TEM images.

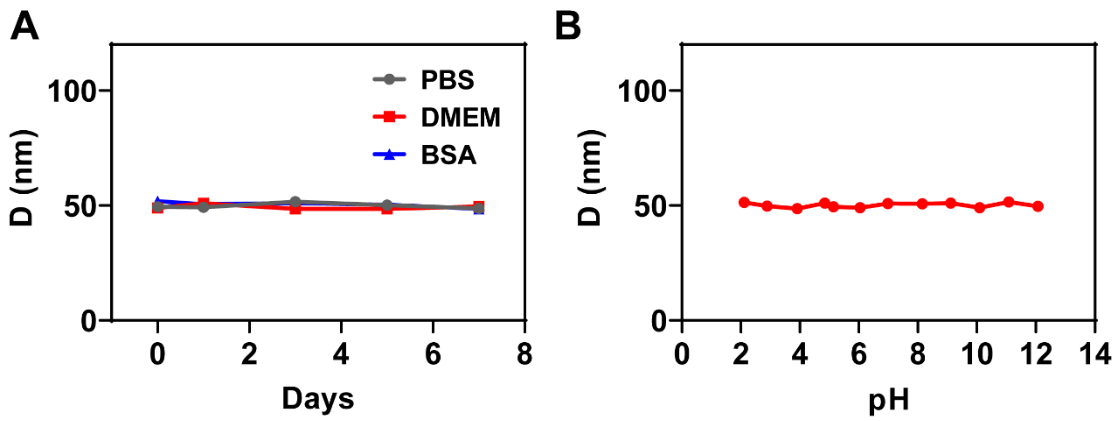

Supplementary Fig. 6. (A) The size of Ir-NP in different solutions over time; (B) The size of Ir-NP in different pH aqueous solutions.

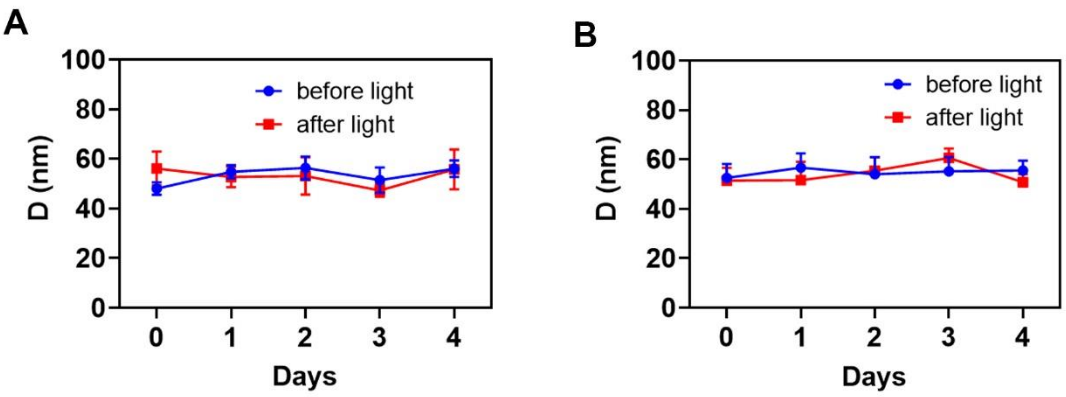

Supplementary Fig. 7. The size of Ir-NP under reducing (A) and hypoxia (B) microenvironment before and after light exposure for 4 days. The reducing microenvironment was induced by adding $Na_2S_2O_4$ (0.1 mM). The statistical data are expressed as mean values ± S.D. (n = 3 independent experiments).

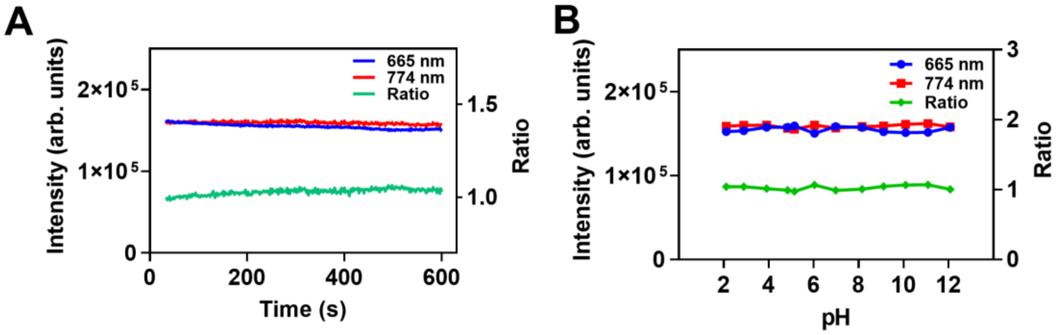

Supplementary Fig. 8. The time (A) and pH (B) photostability of Ir-NP. The term (arb. units) is abbreviated for arbitrary units.

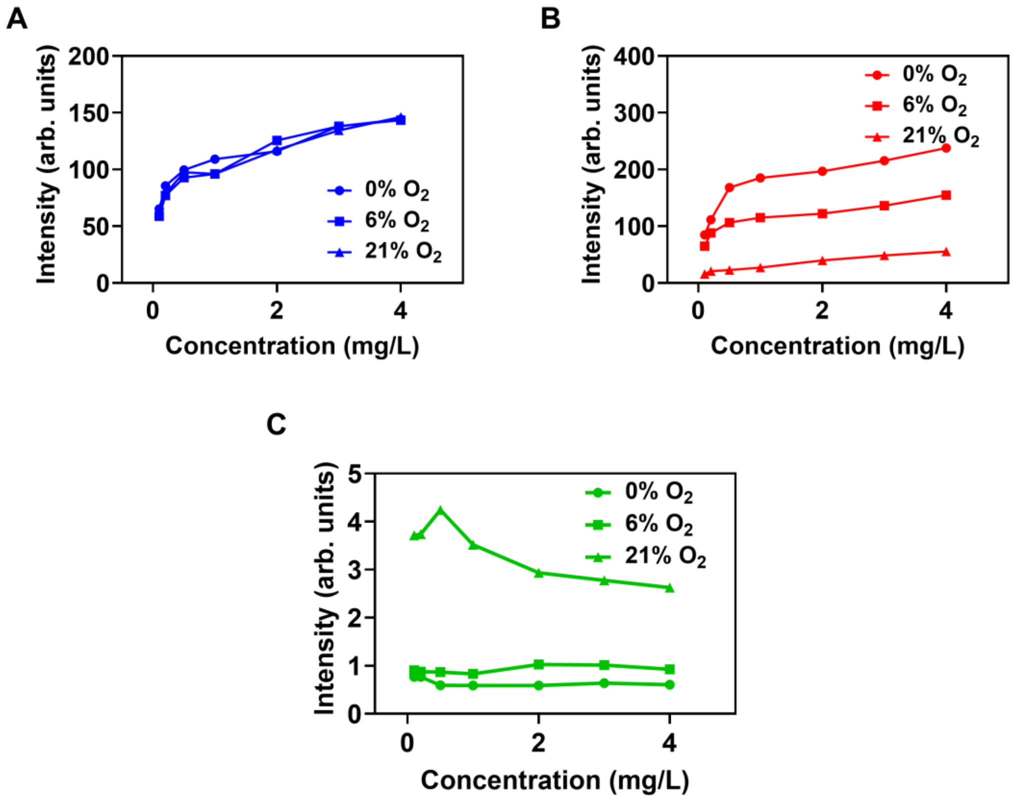

Supplementary Fig. 9. The intensity of Ch1 (A), Ch2 (B) and ratio (Ch1/Ch2, C) in Figure 1G. The term (arb. units) is abbreviated for arbitrary units.

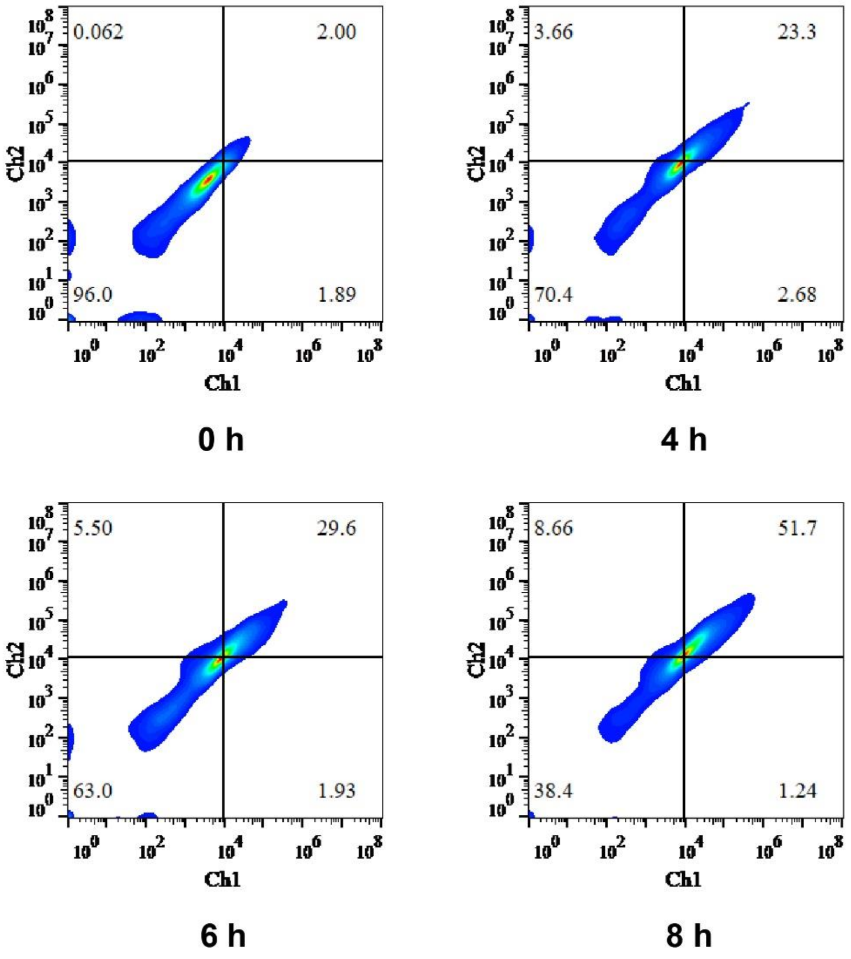

Supplementary Fig. 10. Flow cytometry analysis of the cellular uptake behaviors of Ir-NP at 0 h, 4 h, 6 h and 8 h.

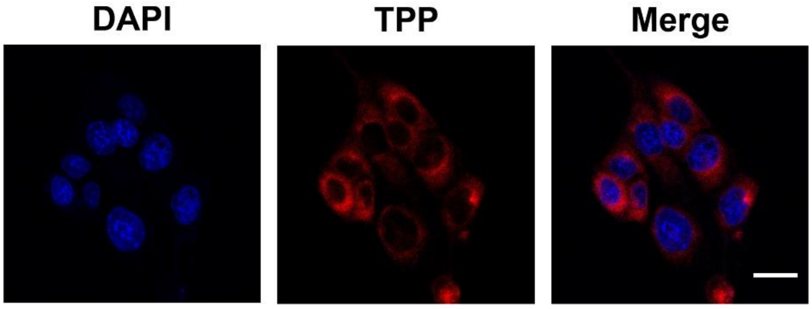

Supplementary Fig. 11. CLSM image of 4T1 cells incubating with Ir-NP for 4 h. Scale bar is 20 μm.

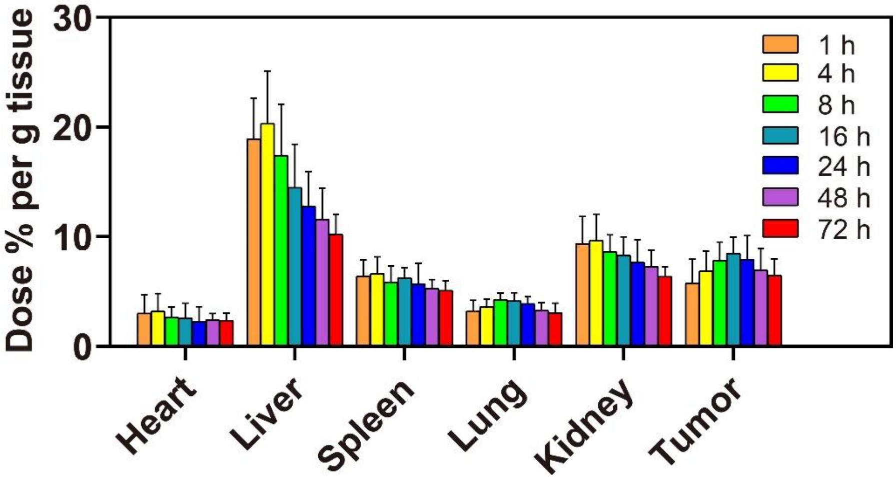

Supplementary Fig. 12. Biodistribution of Ir-NP in different organs at various time points after i.v. injection. The statistical data are expressed as mean values ± S.D. (n = 3 biologically independent animals).

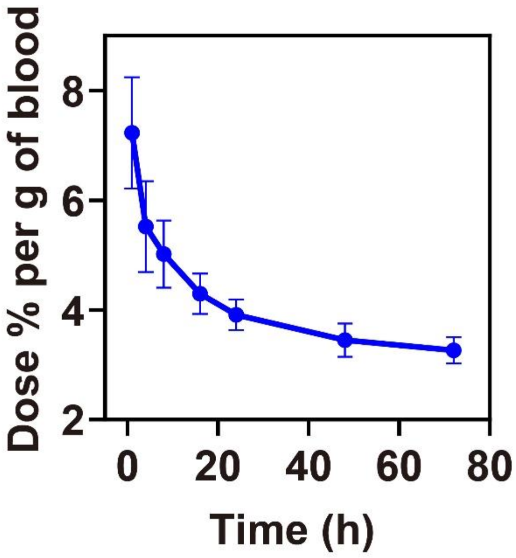

Supplementary Fig. 13. Time-dependent blood clearance profiles of Ir-NP measured in the mice. The statistical data are expressed as mean values ± S.D. (n = 3 biologically independent animals).

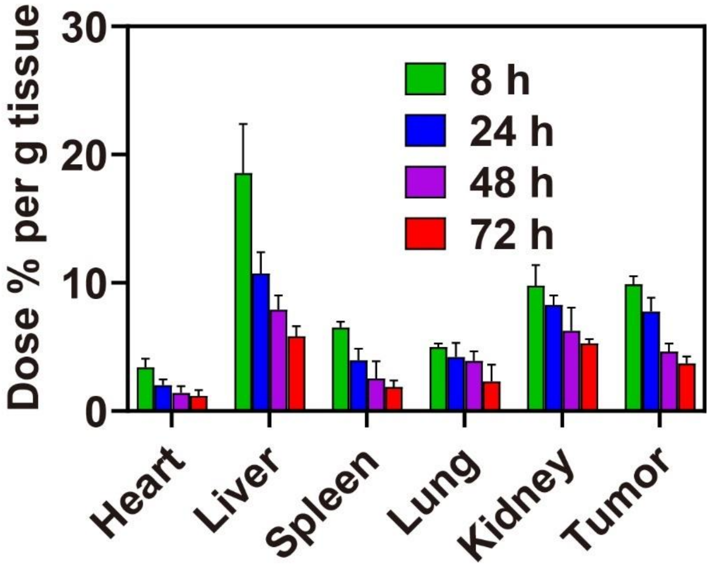

Supplementary Fig. 14. Biodistribution of major organs and tumor tissue at 8, 24, 48, and 72 h post-injection using ICP-AES measurement. The statistical data are expressed as mean values ± S.D. (n = 3 biologically independent animals).

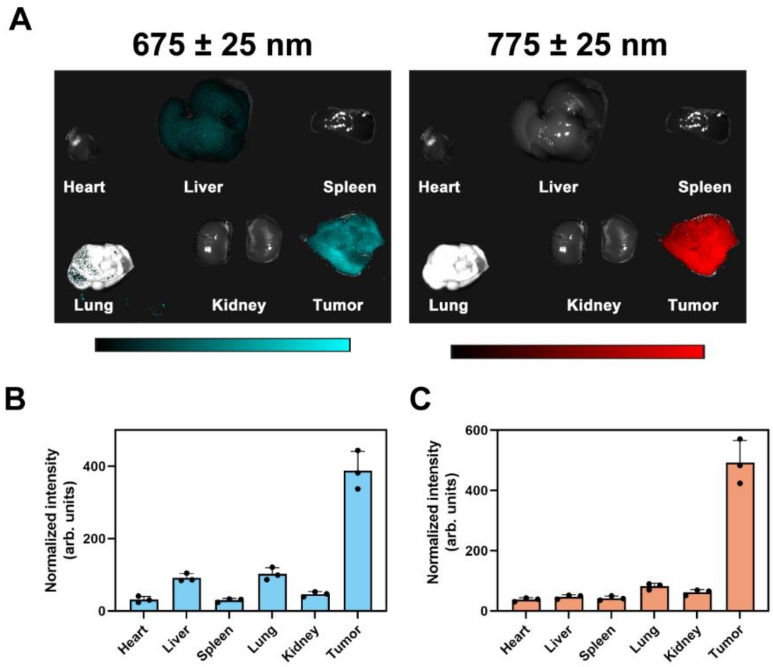

Supplementary Fig. 15. The optical imaging of major organ and tumour from scarified mice. The statistical data are expressed as mean values ± S.D. (n = 3 biologically independent animals).

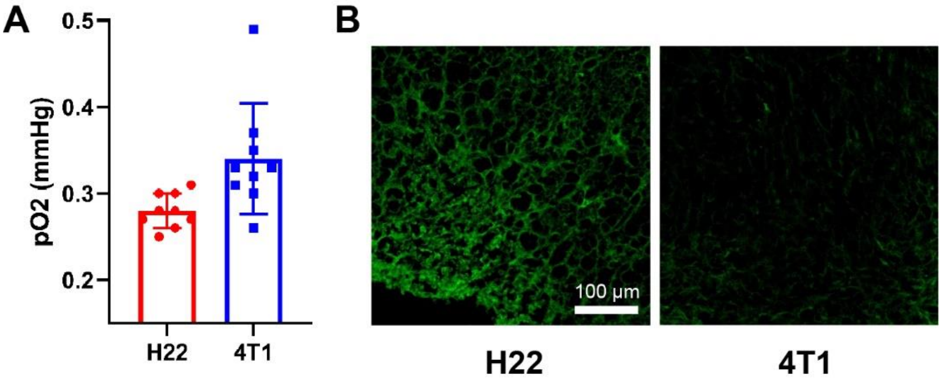

Supplementary Fig. 16. (A) The oxygen concentration of H22 and 4T1 tumours measured by oxygen concentration measurement electrodes. The statistical data are expressed as mean values ± S.D. (n = 9 independent experiments). (B) Frozen sections of the two tumours stained for pimonidazole. Scale bar is 100 μm.

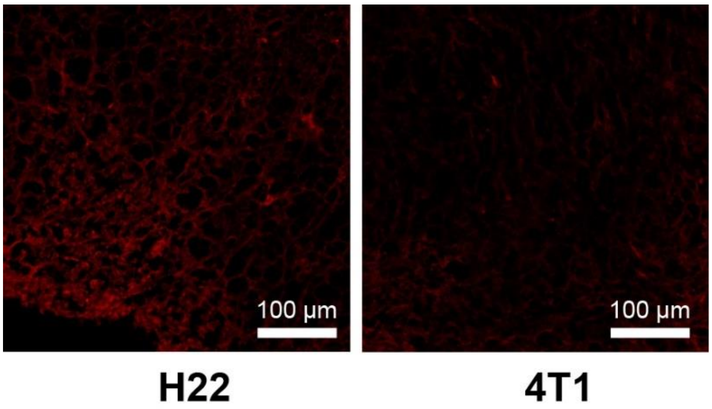

Supplementary Fig. 17. Frozen sections of the two tumours stained for HIF-1α. Scale bar is 100 μm.

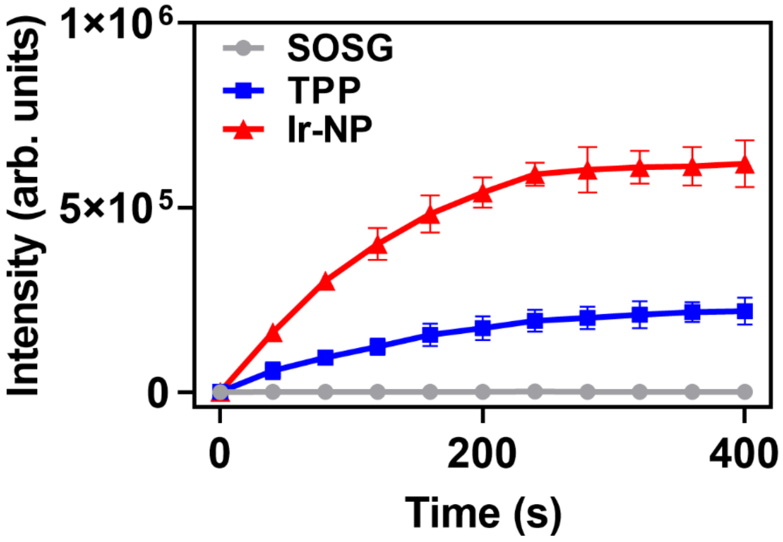

Supplementary Fig. 18. Time related SOSG fluorescence changes in the solution of TPP, Ir-NP and only SOSG under a 630 nm laser. The statistical data are expressed as mean values ± S.D. (n = 3 independent experiments). The term (arb. units) is abbreviated for arbitrary units.

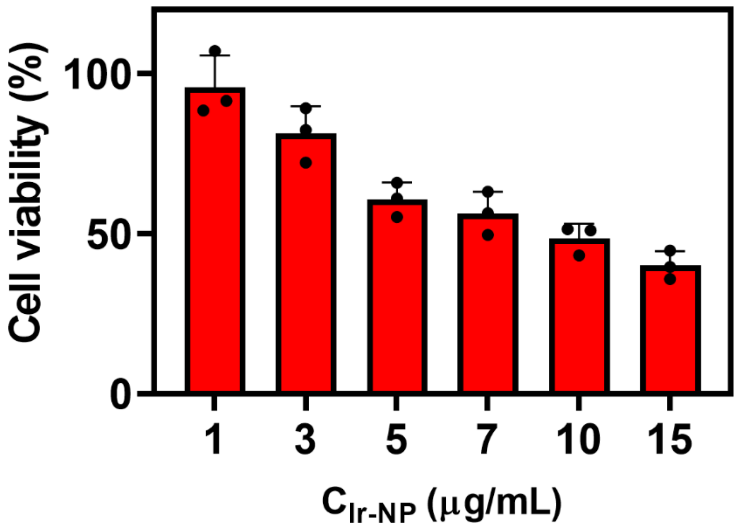

Supplementary Fig. 19. The cell viabilities of 4T1 cells under light irradiation after treatment with Ir-NP. The statistical data are expressed as mean values ± S.D. (n = 3 independent experiments).

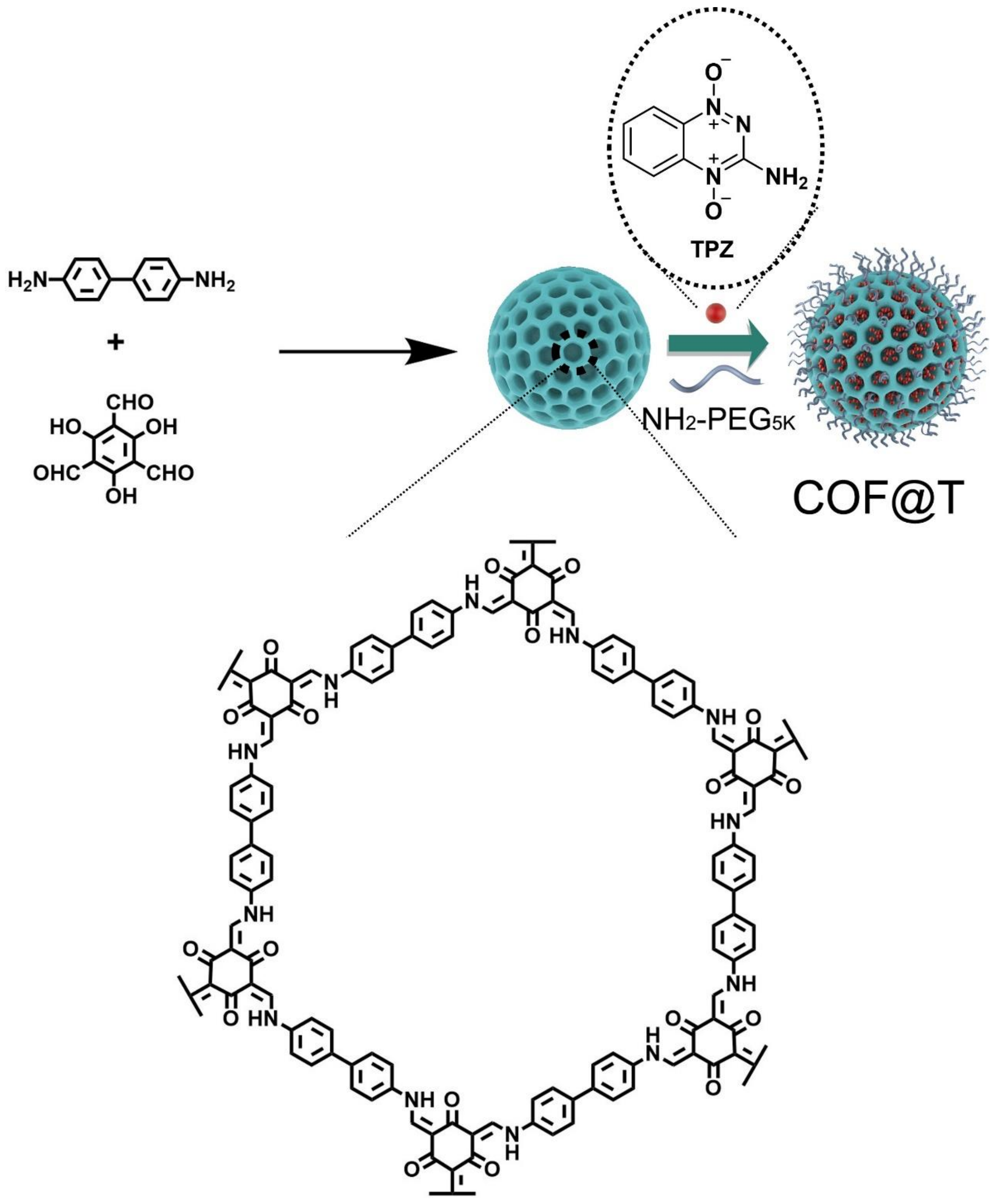

Supplementary Fig. 20. The synthesis route of the COF@T.

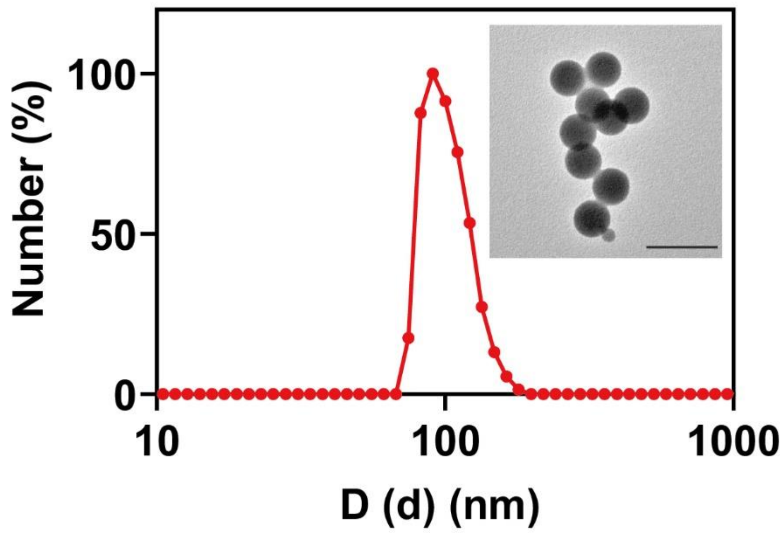

Supplementary Fig. 21. The DLS size distribution and TEM image of the COF.

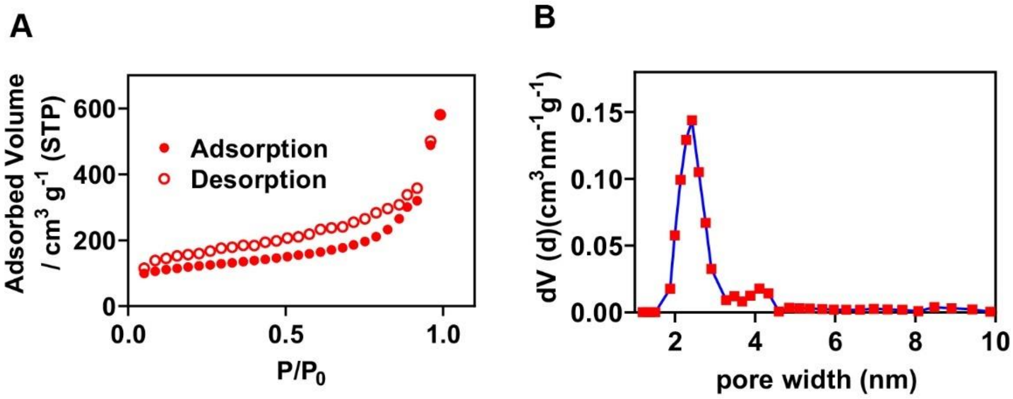

Supplementary Fig. 22. (A) BET surface area measurement of the COF; (B) Pore size distribution of the COF.

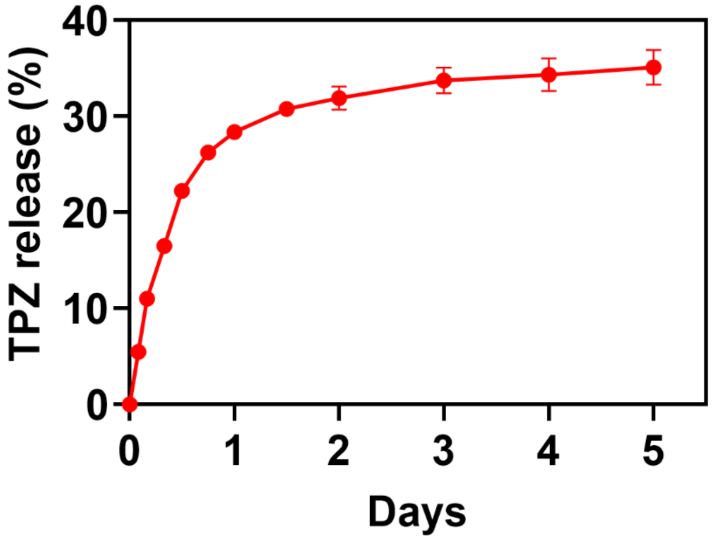

Supplementary Fig. 23. The release curve of TPZ released from COF@T in PBS (pH = 7.4) at 37 °C. The statistical data are expressed as mean values ± S.D. (n = 3 independent experiments).

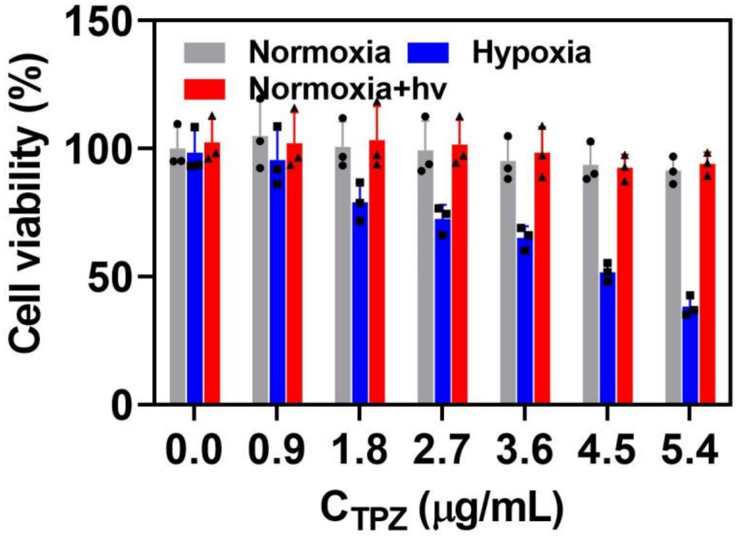

Supplementary Fig. 24. The cell viabilities of 4T1 cells under different conditions after treatment only with different amounts of COF@T. The statistical data are expressed as mean values ± S.D. (n = 3 independent experiments).

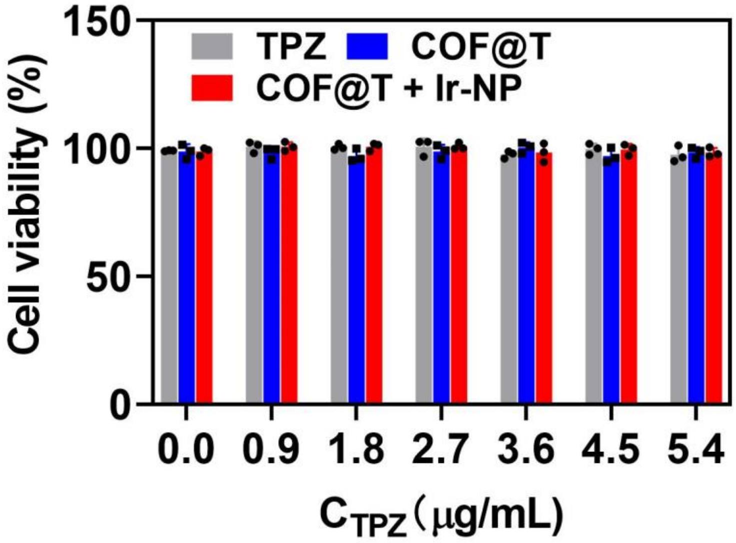

Supplementary Fig. 25. The cell viabilities of Bend.3 cells under normoxia after incubation with different concentration of TPZ, COF@T and COF@T + Ir-NP. The concentration of Ir-NP was 5 mg/L. The statistical data are expressed as mean values ± S.D. (n = 3 independent experiments).

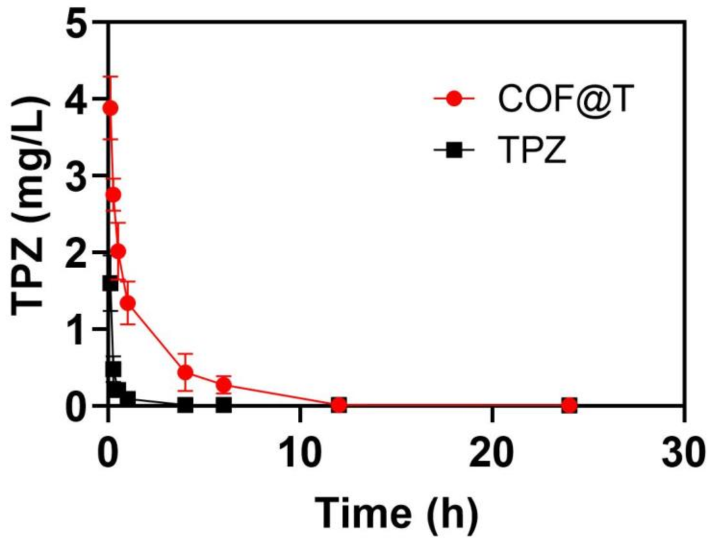

Supplementary Fig. 26. Time-dependent blood clearance profiles of COF@T and TPZ measured in the mice. The statistical data are expressed as mean values ± S.D. (n = 3 biologically independent animals).

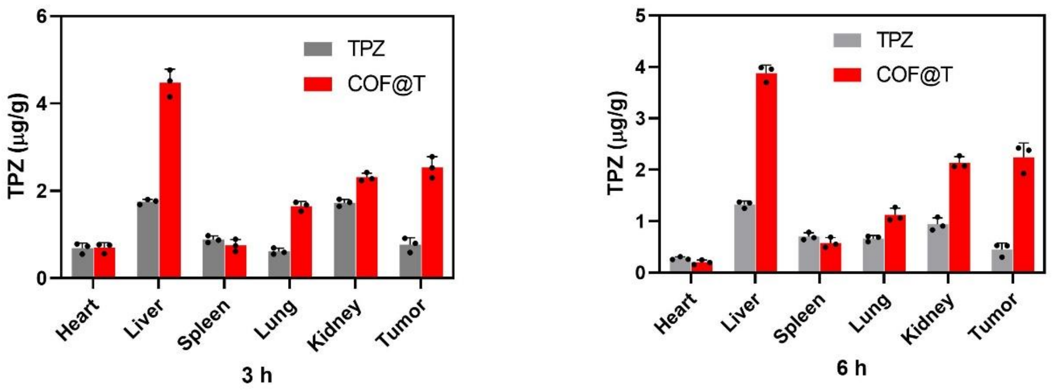

Supplementary Fig. 27. Drug biodistribution in tumour-bearing mice injected with TPZ and COF@T at 3 h and 6 h after injection (i.v.). The statistical data are expressed as mean values ± S.D. (n = 3 biologically independent animals).

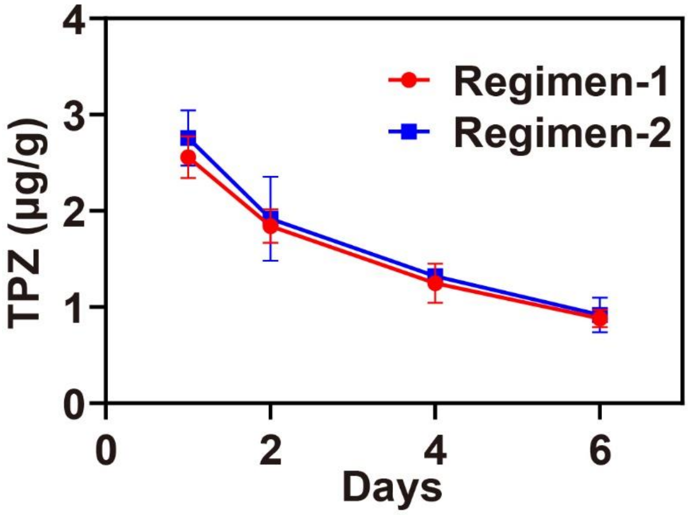

Supplementary Fig. 28. Drug biodistribution in tumor of mice in Regimen-1 and Regimen-2 at different days after injection (i.v.). The statistical data are expressed as mean values ± S.D. (n = 3 biologically independent animals).

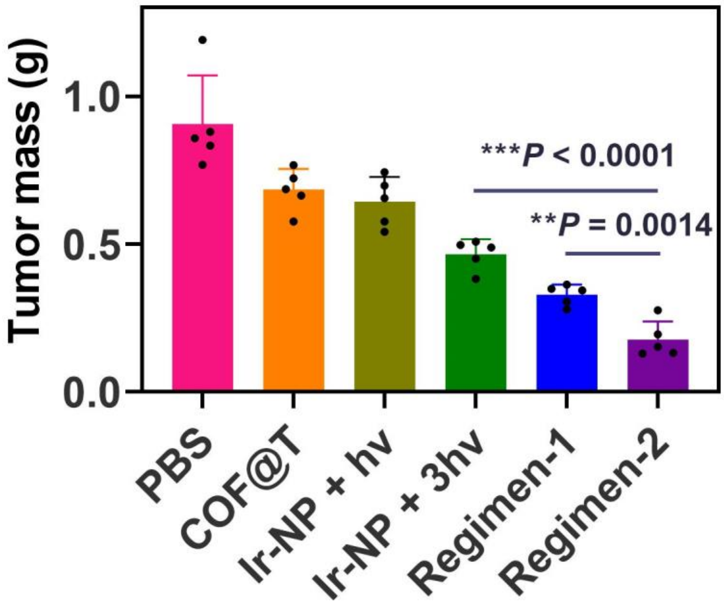

Supplementary Fig. 29. The relative tumour weight of the sacrificed mice at day 14. The statistical data are expressed as mean values ± S.D. (n = 5 biologically independent animals). ***$P < 0.0001$ (Ir-NP + 3hv vs. Regimen-2), **$P < 0.001$ ($P = 0.0014$, Regimen-1 vs. Regimen-2). One-way ANOVA with Dunnett's multiple comparisons test.

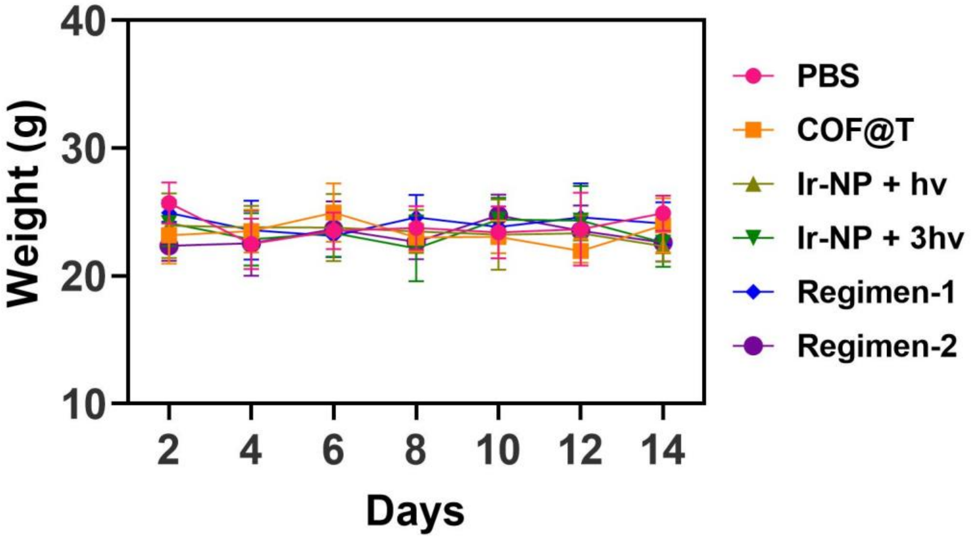

Supplementary Fig. 30. Body weight of mice after different treatments. The statistical data are expressed as mean values ± S.D. (n = 5 biologically independent animals).

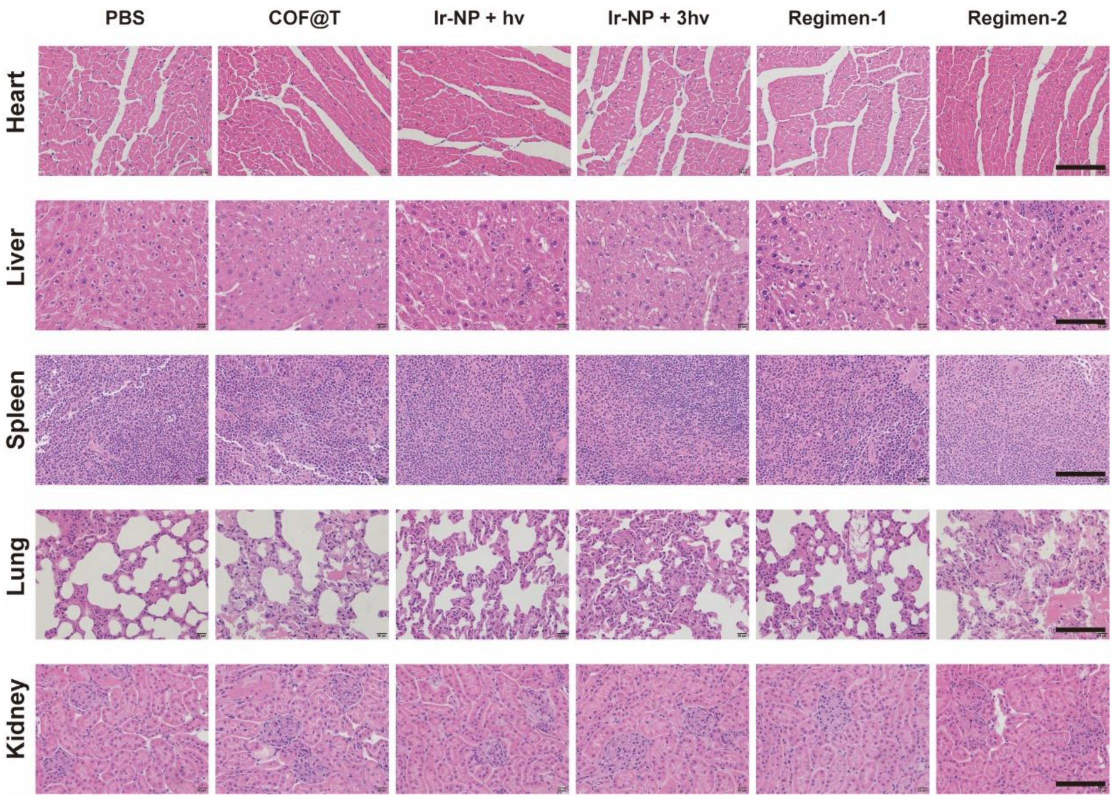

Supplementary Fig. 31. Micrographs of H&E stained main organ slices from mice of different groups collected 14 days after treatments. Scale bar is 100 µm.

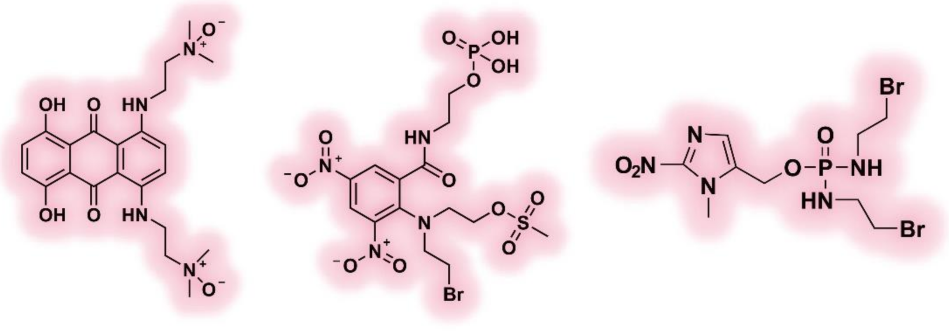

AQ4N **PR-104** **TH-302**

Supplementary Fig. 32. The chemical structure of AQ4N, PR-104 and TH-302.

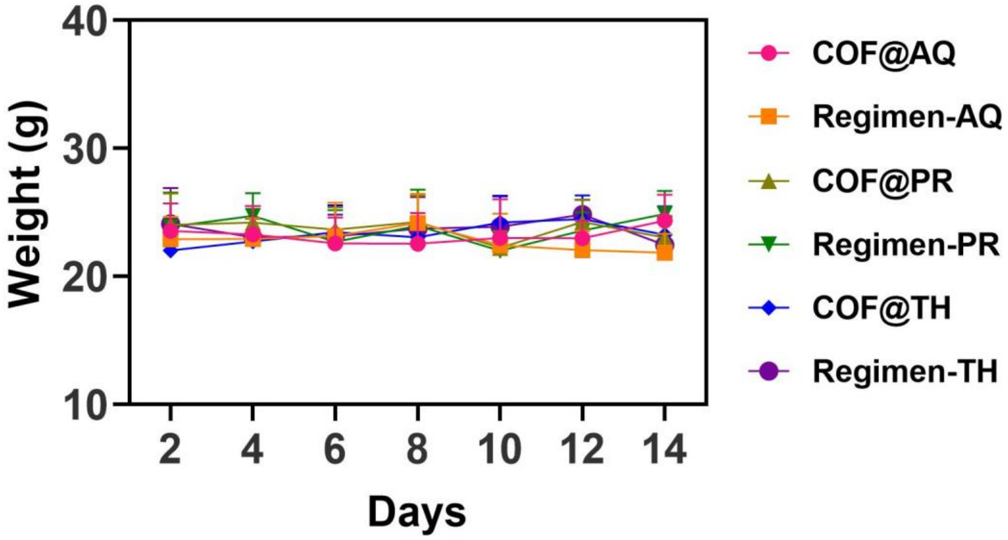

Supplementary Fig. 33. Body weight of mice after different treatments. The statistical data are expressed as mean values ± S.D. (n = 5 biologically independent animals).

Supplementary Table

Mass ratio of chlorosulfonylated IrTBP/TPP	1/20	1/10	1/4	1/2	1/1
I_{665}/I_{774} (21% O_2)	51.22	10.28	1.00	0.36	0.15

Supplementary Table. 1. The ratio of I_{665}/I_{774} after synthesized by different mass ratio of chlorosulfonylated IrTBP to chlorosulfonylated TPP.
