# Supplementary material for: A light-activatable theranostic combination for ratiometric hypoxia imaging and oxygen-deprived drug activity enhancement
Source: Nat Commun. 2024 Jan 2;15:153. doi: 10.1038/s41467-023-44429-y (PMC10762052; doi:10.1038/s41467-023-44429-y)
Supplement: Supplementary file 3 — Reporting Summary [file 41467_2023_44429_MOESM3_ESM.pdf]

nature portfolio

Corresponding author(s): Xiqun Jiang

Last updated by author(s): Nov 30, 2023

Reporting Summary

Nature Portfolio wishes to improve the reproducibility of the work that we publish. This form provides structure for consistency and transparency in reporting. For further information on Nature Portfolio policies, see our Editorial Policies and the Editorial Policy Checklist.

Statistics

For all statistical analyses, confirm that the following items are present in the figure legend, table legend, main text, or Methods section.

n/a	Confirmed	
☐	☒	The exact sample size (*n*) for each experimental group/condition, given as a discrete number and unit of measurement
☐	☒	A statement on whether measurements were taken from distinct samples or whether the same sample was measured repeatedly
☐	☒	The statistical test(s) used AND whether they are one- or two-sided<br>*Only common tests should be described solely by name; describe more complex techniques in the Methods section.*
☒	☐	A description of all covariates tested
☒	☐	A description of any assumptions or corrections, such as tests of normality and adjustment for multiple comparisons
☐	☒	A full description of the statistical parameters including central tendency (e.g. means) or other basic estimates (e.g. regression coefficient) AND variation (e.g. standard deviation) or associated estimates of uncertainty (e.g. confidence intervals)
☐	☒	For null hypothesis testing, the test statistic (e.g. *F*, *t*, *r*) with confidence intervals, effect sizes, degrees of freedom and *P* value noted<br>*Give P values as exact values whenever suitable.*
☒	☐	For Bayesian analysis, information on the choice of priors and Markov chain Monte Carlo settings
☒	☐	For hierarchical and complex designs, identification of the appropriate level for tests and full reporting of outcomes
☒	☐	Estimates of effect sizes (e.g. Cohen's *d*, Pearson's *r*), indicating how they were calculated

Our web collection on statistics for biologists contains articles on many of the points above.

Software and code

Policy information about availability of computer code

Data collection	Fluorescence images were collected by ZEN blue software (version 2.3) and Maestro EX imaging system. Flow-cytometry data were collected by FACS Diva software v6.0.
Data analysis	Fluorescence images were analysed using ImageJ v1.51a Software. NMR spectra were analysed using Mestre Nova LITE v5.2.5-4119 software (Mestre lab Research S.L.). Statistical calculations were performed using GraphPad Prism 8.0. Flow-cytometry results were analysed by FlowJo v10.

For manuscripts utilizing custom algorithms or software that are central to the research but not yet described in published literature, software must be made available to editors and reviewers. We strongly encourage code deposition in a community repository (e.g. GitHub). See the Nature Portfolio guidelines for submitting code & software for further information.

Data

Policy information about availability of data

All manuscripts must include a data availability statement. This statement should provide the following information, where applicable:

- Accession codes, unique identifiers, or web links for publicly available datasets
- A description of any restrictions on data availability
- For clinical datasets or third party data, please ensure that the statement adheres to our policy

The data that support the findings of this study are available from the corresponding author upon request.

Human research participants

Policy information about studies involving human research participants and Sex and Gender in Research.

Reporting on sex and gender	N/A
Population characteristics	N/A
Recruitment	N/A
Ethics oversight	N/A

Note that full information on the approval of the study protocol must also be provided in the manuscript.

Field-specific reporting

Please select the one below that is the best fit for your research. If you are not sure, read the appropriate sections before making your selection.

- [x] Life sciences
- [ ] Behavioural & social sciences
- [ ] Ecological, evolutionary & environmental sciences

For a reference copy of the document with all sections, see nature.com/documents/nr-reporting-summary-flat.pdf

Life sciences study design

All studies must disclose on these points even when the disclosure is negative.

Sample size	All biologically based assays were performed with the usual and sufficient sample size setting determined by previous experiments, such as Nat Commun 12, 2390 (2021); Nat Biomed Eng 1, 0057 (2017) and Nat Commun 6, 5834 (2015). These sample sizes were sufficient for a statistical analysis. All experiments reported have n number and repetitions reported.
Data exclusions	There are no data excluded in this work.
Replication	All experiments were conducted at least three times independently, and similar results were adopted for further analysis to guarantee reproducibility.
Randomization	For in vitro studies, the samples/cells were randomized into different groups prior to treatment. For in vivo experiments, the mice were randomly divided into different groups.
Blinding	Investigators were blinded to group allocation during the experiments.

Reporting for specific materials, systems and methods

We require information from authors about some types of materials, experimental systems and methods used in many studies. Here, indicate whether each material, system or method listed is relevant to your study. If you are not sure if a list item applies to your research, read the appropriate section before selecting a response.

Materials & experimental systems

n/a	Involved in the study	
x		Antibodies
	x	Eukaryotic cell lines
x		Palaeontology and archaeology
	x	Animals and other organisms
x		Clinical data
x		Dual use research of concern

Methods

n/a	Involved in the study	
x		ChIP-seq
	x	Flow cytometry
x		MRI-based neuroimaging

Eukaryotic cell lines

Policy information about cell lines and Sex and Gender in Research

Cell line source(s)	Mouse mammary carcinoma cells 4T1 (a cell line derived from the BALB/c spontaneous mammary carcinoma), mouse hepatoma cells H22, and mouse brain microvascular epithelium cells Bend.3 were purchased from Chinese Academy of Science Cell Bank for Type Culture Collection (Shanghai, China).
Authentication	The cell line was morphologically confirmed according to the information provided by ATCC.
Mycoplasma contamination	The cell line tested for mycoplasma contamination. Mycoplasma contamination was not found.
Commonly misidentified lines (See ICLAC register)	No commonly misidentified cell lines were used.

Animals and other research organisms

Policy information about studies involving animals; ARRIVE guidelines recommended for reporting animal research, and Sex and Gender in Research

Laboratory animals	Female BALB/c mice (4 weeks old) were bought from the Animal center of Drum-tower Hospital. All mice during the experiments were bred in a pathogen-free facility with a 12h light/dark cycle at 20±3 °C and 40–50% humidity and had libitum access to food and water.
Wild animals	The study did not involve wild animals.
Reporting on sex	Sex was not considerated in this study.
Field-collected samples	This study did not involve field-collected samples.
Ethics oversight	All animal experiments were performed in compliance with the protocols approved by the Animal Ethical and Welfare Committee at Nanjing University (Nanjing, China) (Protocol Numbers: IACUC-D2303187).

Note that full information on the approval of the study protocol must also be provided in the manuscript.

Flow Cytometry

Plots

Confirm that:

- [x] The axis labels state the marker and fluorochrome used (e.g. CD4-FITC).
- [x] The axis scales are clearly visible. Include numbers along axes only for bottom left plot of group (a 'group' is an analysis of identical markers).
- [x] All plots are contour plots with outliers or pseudocolor plots.
- [x] A numerical value for number of cells or percentage (with statistics) is provided.

Methodology

Sample preparation	4T1 cells were seeded on 6-well plates. The different Dye or nanoprobe (Ir-NP) were added in the cell containing medium and co-cultured. After collecting the cells (or mitochondrial of cells), the samples were then washed twice and analysed using flow cytometer
Instrument	BD FASCVerse Flow Cytometry
Software	Flowjo_V10
Cell population abundance	Around 10^5 cells were prepared for each sample, and 10^4 cells were analyzed.
Gating strategy	Gating was performed on the FSC-A/SSC-A channels.

- [x] Tick this box to confirm that a figure exemplifying the gating strategy is provided in the Supplementary Information.
